# The effect of model complexity on the human center of mass estimation using the statically equivalent serial chain technique

**DOI:** 10.1038/s41598-023-47337-9

**Published:** 2023-11-20

**Authors:** Elie Chebel, Burcu Tunc

**Affiliations:** 1 Department of Computer Engineering, Bahcesehir University, Istanbul, 34353 Turkey; 2 Department of Biomedical Engineering, Bahcesehir University, Istanbul, 34353 Turkey

**Keywords:** Biomedical engineering, Rehabilitation

## Abstract

Estimating the human center of mass (CoM) has long been recognized as a highly complex process. A relatively recent and noteworthy technique for CoM estimation that has gained popularity is the statically equivalent serial chain (SESC). This technique employs a remodeling of the human skeleton as a serial chain where the end effector represents the CoM location. In this study, we aimed to evaluate the impact of model complexity on the estimation capability of the SESC technique. To achieve this, we designed and rigorously assessed four distinct models with varying complexities against the static center of pressure (CoP) as reference, by quantifying both the root-mean-square (RMS) and correlation metrics. In addition, the Bland–Altman analysis was utilized to quantify the agreement between the estimations and reference values. The findings revealed that increasing the model complexity significantly improved CoM estimation quality up to a specific threshold. The maximum observed RMS difference among the models reached 9.85 mm. However, the application and task context should be considered, as less complex models still provided satisfactory estimation performance. In conclusion, the evaluation of model complexity demonstrated its impact on CoM estimation using the SESC technique, providing insights into the trade-off between accuracy and complexity in practical applications.

## Introduction

The human center of mass (CoM) location has been established as an essential tool in various areas of human related sciences, ranging from rehabilitation and physical therapy^1,2^ to athletes training and evaluation^3^. However, knowing the exact CoM location is far from an easy task, especially when it comes to a complex structure such as the human body.

Numerous methods have been proposed throughout the decades to estimate the CoM location in humans. These methods can be divided into two main categories: force measurement and kinematic methods. Those which rely on force measurement are typically implemented using a force platform which measures the ground reaction forces (GRF) and calculates the center of pressure (CoP) location. For example, by removing high-frequency components from CoP measurements, Caron et al.^4^ managed to estimate the human CoM’s ground projection. The determination of the CoM trajectory in this approach is done by applying the low-pass filter to the CoP frequency content. The design of their filter was based on the previous work done by Brenière^5^, where a clear relationship between CoM and CoP oscillations was found.

The other common approach is using CoP readings from a force platform along with Newton’s equations to compute the CoM location. By utilizing the double integration of the acceleration, calculated from the GRF, the CoM’s position achieved to be calculated^6,7^. This method has been considered as the gold standard for CoM estimation in some studies^8^ and used as a reference technique when comparing other methods^9^. Lafond et al.^10^ described it as an attractive method in the clinical perspective and only requires a force platform to quantify the postural control under various somatosensory conditions. However, the use of this method does come with several limitations, such as determining the initial integration constants—initial position and initial velocity—and being very difficult to apply in real-time due to the necessity of filtering the noisy measurements^11^.

As for tracing the human CoM using kinematic methods, the process mainly relies on using a tracking device that can estimate the position(s) and orientation(s) of a desired part or parts of the body. The sacral marker method, for example, is one of the simplest approaches adopted in CoM trajectory estimations. According to Braune and Fischer^12^, the CoM is placed inside or closely behind the second sacral vertebra while a person is in a standing posture. In this sense, only tracking the position of this vertebra is deemed enough to track the CoM. An enhancement to this method was proposed by Floor-Westerdijk et al.^13^, in which they stated that using the trunk’s orientation can improve the approximation’s accuracy. One of the main benefits of such a method is the low cost of equipment needed for the process, as it only requires the use of one reflective marker or one inertial measurement unit (IMU). On the other hand, the use of this method is limited to CoM estimation for only few tasks such as certain pathological gaits^14^ or quiet standing and would produce a very inaccurate estimation in otherwise tasks. In certain tasks like squat or bending, for example, the CoM can be located outside the human body, thus the measured location of this vertebra will give a false estimation of the CoM’s location. Moreover, the adoption of a single inertial measurement unit (IMU) remains an unreliable solution, as recent research highlights significant error fluctuations, particularly in tasks involving high rotation rates^15^.

The other commonly used method in CoM estimation is the segmental analysis method. In this technique, the human body is divided into a specific number of segments, and anthropometric data are assigned to each segment including the approximate mass and segment-CoM location. These approximations are usually taken from anthropometric tables previously published in the literature. The most commonly used tables are those published by De Leva^16^, Winter^17^ and Zatsiorsky et al.^18^. This method may be practical in some cases, but the implementation of such tables in studies involving other types of populations has shown large CoM estimation inaccuracy. Other studies attempted to determine the CoM or mass of individual limbs on living subjects^12,16,17,19^. The protocol followed in these studies was to make the person lie down on a balance board and determine the limbs’ parameters during various postures by measuring the reaction moments. This determination of each subject’s specific parameters can be very time-consuming and still prone to error that leads to large estimation inaccuracies. Another kinematic-based technique is the statically equivalent serial chain (SESC). This particular technique proposed by Cotton et al.^20^, based on the work previously done by Espiau and Boulic^21^ on CoM estimation in robotics, presents a way to estimate the CoM of a branched-chain without the need of anthropometric measurements. Rather than assigning the anthropometric data to each segment of the body, an identification phase has to be carried out prior to the experimentation phase.

This identification phase provides a subject-specific vector of parameters equivalent to the anthropometric data needed to estimate the CoM as in the segmentation method. SESC is credited to overcoming the challenges imposed by atypical body types when it comes to CoM estimation.

Throughout the literature, SESC has been widely used in different types of CoM estimation studies, as it has been utilized in studies conducted on different populations, such as healthy and elderly^22^, stroke survivors^23^, and obese^24^, as well as in robotics^20,25^ and computer simulations^26^. All the studies that have compared SESC to the segmental analysis method have shown that SESC provides more accurate estimation. Some of the studies proposed enhancements to SESC as well. Li et al.^27^ proposed modifying the matrix containing the joint angle information to avoid having a non-unique solution while solving the equation for SESC parameters. González et al.^28^ proposed an additional reduction of the model, taking into consideration the same geometrical aspects and the symmetry of certain limbs. As a result, the subject-specific SESC vector can be further simplified, leading to a reduction in the number of postures required for identification. Almandeel et al.^29^ introduced another form of SESC modeling that relies on a node-based articulated system instead of joints. This way of modeling is said to reduce the processing time needed for real-time SESC application by eliminating the time needed for joint angles calculations. The most recent improvement of SESC was introduced by González et al.^30^, in which they presented a mixed SESC identification process that includes non-static postures, implemented using a Kalman filter that allows automatic switching between static and dynamic models.

Taking all the advantages which SESC offers over other methods, it became an attractive tool for human CoM estimation, however, choosing the best kinematic structure for SESC modeling is not a straightforward decision. Designing a humanoid multibranched chain model that represents the kinematic model of a human is a key part of the SESC’s implementation, as the design of the SESC model varies according to the number of segments, the number of joints used, and the degrees of freedom (DoF) of each joint. Throughout the literature, there have been a wide variety of SESC models presented, while some used similar biomechanical representations of the human body, others chose different skeletal structures. The complexities of those models ranged from a considerably abstract representation, comprised of three segments and three joints with three DoF^22^, to highly complex models comprised of 9 segments and 9 joints with 19 DoF^28^. However, there have been no comparisons mentioned in the literature between SESC models with different structural complexities. For the purpose of investigating the relationship between the model complexity and the estimation accuracy of SESC, in this study, a comparison between four different models is implemented using four different biomechanical structures. Those structures were purposely designed with different number of joints, different joint sets, and different joint ranges of motion limited by the number of DoFs used. Using the same collected dataset, we rigorously assessed these SESC models by evaluating their estimation capabilities against a common reference method, conducting a comprehensive evaluation that primarily focused on examining error differences, accuracy, precision, and detecting biases.

## Methods

### Human biomechanical models

**Figure 1:**
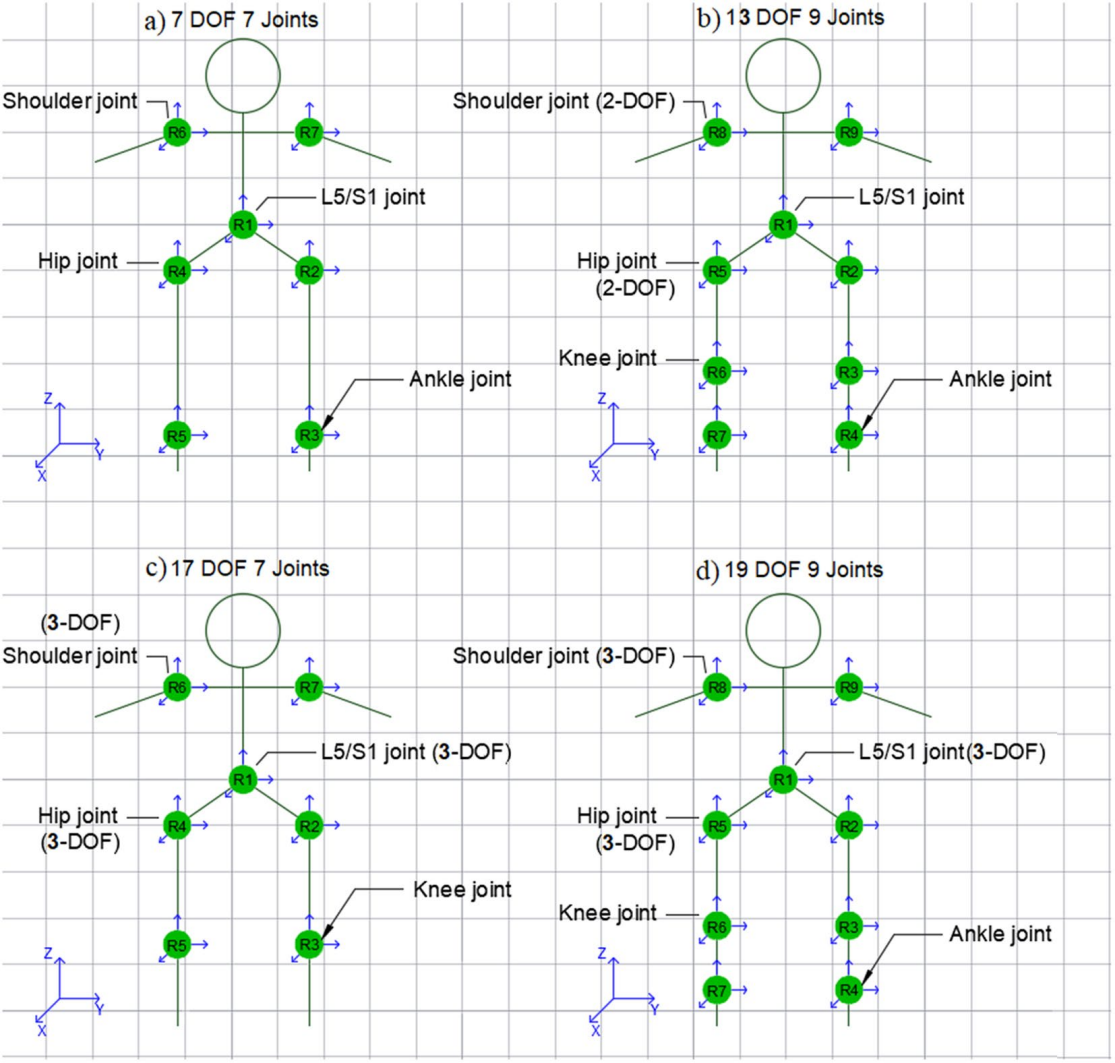
The four different branched-chain structures which were designed for this study, are alphabetically labeled according to complexity increase.

Representing the human body as a biomechanical system can be a very complex process. The motions of human joints are usually approximated by typical mechanical joints. Shoulder and hip joints, for example, are often defined as ball and socket joints, while other joints with a lower range of motion like elbow and knee joints are represented as hinge joints. Building upon these approaches, we designed four different branched-chain structures for this study (Fig. 1). The designed structures vary according to the number of segments, joints, and DoF used in each joint. The following models are labeled alphabetically a, b, c, and d, with “a” being the least complex structure and “d” being the most complex.

The CoM of a branched chain comprised of N-segments is calculated as the weighted sum of each segment as shown in Eq. (1):

1 $$\begin{aligned} \overrightarrow{CoM}=\frac{\sum _{i=1}^{N} m_{i} \cdot \vec {C}_{i}^{G}}{M}, \end{aligned}$$

where $\overrightarrow{CoM}$ is a 3-by-1 vector representing the 3D CoM location with respect to the global frame G, $m_{i}$ is the mass of each segment, $\vec {C}_{i}^{G}$ is the location of each segment’s CoM with respect to G, and M is the total mass of the structure $\textit{M} = \sum _{i=1}^{N} m_{i}$.

Calculating $\vec {C}_{i}^{G}$ for each segment of a branched chain requires a series of matrix multiplications (homogenous transformation matrices). The following paragraphs present a detailed expansion of the Eq. (1) according to each one of the models which are shown in Fig. 1.

The simplest model designed, model (a), is a branched-chain with 7 segments connected using 7 joints each with only 1 DoF. The joints selected for this model were: lumbosacral joint (L5/S1), shoulders, and ankles. The CoM of this structure can be calculated using the following equation:

2 $$\begin{aligned} \begin{array}{r} \left\{ \begin{array}{c} {\overrightarrow{CoM}}(a) \\ 1 \end{array}\right\} =\frac{m_1}{M} H_1\left\{ \begin{array}{c} \vec {C}_1 \\ 1 \end{array}\right\} +\frac{m_2}{M} H_1 H_2\left\{ \begin{array}{c} \vec {C}_2 \\ 1 \end{array}\right\} +\frac{m_3}{M} H_1 H_2 H_3\left\{ \begin{array}{c} \vec {C}_3 \\ 1 \end{array}\right\} +\frac{m_4}{M} H_1 H_4\left\{ \begin{array}{c} \vec {C}_4 \\ 1 \end{array}\right\} \\ +\frac{m_5}{M} H_1 H_4 H_5\left\{ \begin{array}{c} \vec {C}_5 \\ 1 \end{array}\right\} +\frac{m_6}{M} H_1 H_6\left\{ \begin{array}{c} \vec {C}_6 \\ 1 \end{array}\right\} +\frac{m_7}{M} H_1 H_7\left\{ \begin{array}{c} \vec {C}_7 \\ 1 \end{array}\right\} \end{array}, \end{aligned}$$

where, $$H_i= \left[ \begin{array}{cc} R_1 &{} \vec {d}_1 \\ 0 &{} 1 \end{array}\right]$$ is a 4-by-4 homogenous transformation matrix linking the frame *i-1* to the frame *i*, containing a 3-by-3 rotation matrix $R_i$ and a displacement vector $d_i$. The angles recorded for all joints in this model were flexion/extension angles $'\alpha _i'$ (described by a rotation around the Y-axis) which makes

$$\begin{aligned} R_i= R_y (\alpha ) = \left[ \begin{array}{ccc} \cos (\alpha ) &{} 0 &{} \sin (\alpha ) \\ 0 &{} 1 &{} 0 \\ -\sin (\alpha ) &{} 0 &{} \cos (\alpha ) \end{array}\right] \ \end{aligned}$$

Model (b) on the other hand, is comprised of 9 segments connected by 9 joints with a total number of 13 DoFs. In this model, L5/S1, knee, and ankle joints are defined as hinge joints (1 DoF) with a flexion/extension motion thus the rotation matrices of these joints are similar to $R_y$ used in the previous model, while the shoulders and hips joints are defined as universal joints (2 DoF), representing both flexion/extension and adduction/abduction motions, and their motion is described by the following rotation sequence:

$$\begin{aligned} R_i= R_{xy} = R_y (\alpha )R_x (\beta ) = \left[ \begin{array}{ccc} \cos (\alpha ) &{} 0 &{} \sin (\alpha ) \\ 0 &{} 1 &{} 0 \\ -\sin (\alpha ) &{} 0 &{} \cos (\alpha ) \end{array}\right] \left[ \begin{array}{ccc} 1 &{} 0 &{} 0 \\ 0 &{} \cos (\beta ) &{} -\sin (\beta ) \\ 0 &{} \sin (\beta ) &{} \cos (\beta ) \end{array}\right] . \end{aligned}$$

The equation representing the CoM of the model (b) is:

3 $$\begin{aligned} \begin{array}{r} \left\{ \begin{array}{c} \overrightarrow{CoM}(b) \\ 1 \end{array}\right\} =\frac{m_1}{M} H_1\left\{ \begin{array}{c} \vec {C}_1 \\ 1 \end{array}\right\} +\frac{m_2}{M} H_1 H_2\left\{ \begin{array}{c} \vec {C}_2 \\ 1 \end{array}\right\} +\frac{m_3}{M} H_1 H_2 H_3\left\{ \begin{array}{c} \vec {C}_3 \\ 1 \end{array}\right\} +\frac{m_4}{M} H_1 H_2 H_3 H_4\left\{ \begin{array}{c} \vec {C}_4 \\ 1 \end{array}\right\} \\ +\frac{m_5}{M} H_1 H_5\left\{ \begin{array}{c} \vec {C}_5 \\ 1 \end{array}\right\} +\frac{m_6}{M} H_1 H_5 H_6\left\{ \begin{array}{c} \vec {C}_6 \\ 1 \end{array}\right\} +\frac{m_7}{M} H_1 H_5 H_6 H_7\left\{ \begin{array}{c} \vec {C}_7 \\ 1 \end{array}\right\} \\ +\frac{m_8}{M} H_1 H_8\left\{ \begin{array}{c} \vec {C}_8 \\ 1 \end{array}\right\} +\frac{m_9}{M} H_1 H_9\left\{ \begin{array}{c} \vec {C}_9 \\ 1 \end{array}\right\} \end{array}. \end{aligned}$$

As for the third representation, model (c) is designed to have the same number of segments as the first model (7 segments), but with a knee joint instead of the ankle joint, and a total of 17 DoF compared to 7 DoF in model (a). This increase in DoF is due to considering the shoulder, hip, and L5/S1 joints as ball and socket joints, each having 3 DoFs. It should be noted that in the case of Xsens, the motion capture (mocap) system used in this study, the joint angles are extracted following the ZXY ‘fixed’ angles sequence. In other words, the matrix representing the rotation of a spherical joint (for example, the shoulder joint in models c and d) would be equal to the following matrix multiplications $R_{zxy}=R_y (\alpha )R_x(\beta )R_z(\gamma )$.

The rotation matrix defining the 3 DoFs is calculated as follows:

$$\begin{aligned} R_i= R_{zxy} = R_y(\alpha )R_x(\beta )R_z(\gamma ) = \left[ \begin{array}{ccc} \cos (\alpha ) &{} 0 &{} \sin (\alpha ) \\ 0 &{} 1 &{} 0 \\ -\sin (\alpha ) &{} 0 &{} \cos (\alpha ) \end{array}\right] \left[ \begin{array}{ccc} 1 &{} 0 &{} 0 \\ 0 &{} \cos (\beta ) &{} -\sin (\beta ) \\ 0 &{} \sin (\beta ) &{} \cos (\beta ) \end{array}\right] \left[ \begin{array}{ccc} \cos (\gamma ) &{} -\sin (\gamma ) &{} 0 \\ \sin (\gamma ) &{} \cos (\gamma ) &{} 0 \\ 0 &{} 0 &{} 1 \end{array}\right] . \end{aligned}$$

The CoM of model (c) can be calculated using the following equation:

4 $$\begin{aligned} \begin{array}{r} \left\{ \begin{array}{c} {\overrightarrow{CoM}}(c) \\ 1 \end{array}\right\} =\frac{m_1}{M} H_1\left\{ \begin{array}{c} \vec {C}_1 \\ 1 \end{array}\right\} +\frac{m_2}{M} H_1 H_2\left\{ \begin{array}{c} \vec {C}_2 \\ 1 \end{array}\right\} +\frac{m_3}{M} H_1 H_2 H_3\left\{ \begin{array}{c} \vec {C}_3 \\ 1 \end{array}\right\} +\frac{m_4}{M} H_1 H_4\left\{ \begin{array}{c} \vec {C}_4 \\ 1 \end{array}\right\} \\ +\frac{m_5}{M} H_1 H_4 H_5\left\{ \begin{array}{c} \vec {C}_5 \\ 1 \end{array}\right\} +\frac{m_6}{M} H_1 H_6\left\{ \begin{array}{c} \vec {C}_6 \\ 1 \end{array}\right\} +\frac{m_7}{M} H_1 H_7\left\{ \begin{array}{c} \vec {C}_7 \\ 1 \end{array}\right\} \end{array}. \end{aligned}$$

Similarly, model (d) shares the same number of segments and joints as the model (b) and has the same joint modifications applied for model (c) regarding the shoulder, hip, and L5/S1 joints. The total number of DoF for model (d) is 19 DoFs. In other words, the rotation matrices used for these joints follow the same $R_{zxy}$ described above. The equation representing the CoM of the model (d) is:

5 $$\begin{aligned} \begin{array}{r} \left\{ \begin{array}{c} {\overrightarrow{CoM}}(d) \\ 1 \end{array}\right\} =\frac{m_1}{M} H_1\left\{ \begin{array}{c} \vec {C}_1 \\ 1 \end{array}\right\} +\frac{m_2}{M} H_1 H_2\left\{ \begin{array}{c} \vec {C}_2 \\ 1 \end{array}\right\} +\frac{m_3}{M} H_1 H_2 H_3\left\{ \begin{array}{c} \vec {C}_3 \\ 1 \end{array}\right\} +\frac{m_4}{M} H_1 H_2 H_3 H_4\left\{ \begin{array}{c} \vec {C}_4 \\ 1 \\ \end{array}\right\} \\+ \frac{m_5}{M} H_1 H_5\left\{ \begin{array}{c} \vec {C}_5 \\ 1 \end{array}\right\} +\frac{m_6}{M} H_1 H_5 H_6\left\{ \begin{array}{c} \vec {C}_6 \\ 1 \end{array}\right\} +\frac{m_7}{M} H_1 H_5 H_6 H_7\left\{ \begin{array}{c} \vec {C}_7 \\ 1 \end{array}\right\}\\ + \frac{m_8}{M} H_1 H_8\left\{ \begin{array}{c} \vec {C}_8 \\ 1 \end{array}\right\} +\frac{m_9}{M} H_1 H_9\left\{ \begin{array}{c} \vec {C}_9 \\ 1 \end{array}\right\} \end{array}. \end{aligned}$$

### SESC mathematical modeling

The CoM in SESC is calculated as the end-effector of a serially linked chain. The transition from the traditional equation form of CoM calculation (as in Eq. 1) to the manipulated form of a serial chain is fully described in Cotton et al.^20^. The equation derived by that study to calculate the CoM’s location is shown below.

6 $$\begin{aligned} \overrightarrow{CoM}= \vec {d_1} + \left[ \begin{array}{lllll} R_{1}&\cdots&R_{n} \end{array}\right] \left[ \begin{array}{c} \overrightarrow{v_{1}} \\ \vdots \\ \overrightarrow{v_{n}} \end{array}\right] =\vec {d_1} + B\vec {V}, \end{aligned}$$

where $\overrightarrow{d_1}$ is the location of the root joint of the model, $R_{1\rightarrow n}$ are the rotation matrices containing the angles values of each joint, and $\vec {v}_{1\rightarrow n}$ are 3-by-1 vectors containing the subject specific inertial parameters ($m_i$, $\overrightarrow{d_i}$ and *M*). It should be noted that all components of the SESC vector $\overrightarrow{V}$ are constants and do not change during motion of the structure. In the case of a floating base system, the vector $\overrightarrow{d_1}$, which is the displacement of the first joint with respect to the global frame, is not constant, so the Eq. (6) is further modified in the following manner:

7 $$\begin{aligned} \overrightarrow{CoM} - \vec {d_1} = B\vec {V} = \overrightarrow{CoM'}. \end{aligned}$$

To calculate the SESC vector, in an ideal situation where the 3D CoM locations and their corresponding joint angles are known, and with *enough number of configurations* collected, the constant vector $\vec {V}$ can be calculated using the Moore–Penrose pseudoinverse as shown in Eq. (8). The employment of the pseudoinverse ensures that a solution exists even when the matrix is non-square. This advantage makes it a pertinent approach for the computation of the vector $\vec {V}$.

8 $$\begin{aligned} \vec {V}=B^{\dagger }\overrightarrow{CoM}, \end{aligned}$$

where

9 $$\begin{aligned} B^{\dagger } = (B^TB)^{-1}B^T. \end{aligned}$$

Computing $\vec {V}$ using this method will result in the best fit solution to our system of linear equations (Penrose 1955).

For situations in which the CoM’s location is partially known, the process of SESC identification can still be applied in the same manner but with a few modifications of the used equations. For example, Cotton and his colleagues in their studies which included human subjects^22^, regarded the CoP collected using a force platform during quiet standing as an approximation of the projection of the CoM onto the ground.

By using this information, during this phase, the 3rd row in B can be eliminated and the Eq. (8) becomes:

10 $$\begin{aligned} \vec {V}=\left[ \begin{array}{c} B_x \\ B_y \end{array}\right] ^{\dagger }\left[ \begin{array}{c} CoP_x - d_{1,x} \\ CoP_y - d_{1,y} \end{array}\right] = B'^{\dagger } \overrightarrow{CoP'}. \end{aligned}$$

To ensure that B’ is invertible by the pseudo-inverse method, the total number of rows should exceed the number of columns. The number of columns in this matrix is equal to three times the number of joints (n) in the system. Therefore, if the partial CoM information is extracted from a 2D plane ($CoP_x$ and $CoP_y$ for example), at least a total number of (3n/2) different static postures measurements (k) should be collected. Thus, the following form of equation is created:

11 $$\begin{aligned} \vec {V}=\left[ \begin{array}{c} \mathrm {{B}}_{x, \textrm{1}} \\ \mathrm {{B}}_{x, \textrm{1}} \\ \vdots \\ \mathrm {{B}}_{x, m} \\ \mathrm {{B}}_{x, {m}} \end{array}\right] ^{\dagger }\left[ \begin{array}{c} {\text {CoP}}_{x, 1}^{\prime } \\ {\text {CoP}}_{y, 1}^{\prime } \\ \vdots \\ {\text {CoP}}_{x, m}^{\prime } \\ {\text {CoP}}_{y, m}^{\prime } \end{array}\right] . \end{aligned}$$

After $\vec {V}$ is identified, the full 3D CoM can be calculated with only joint angles values by using Eq. (7).

### Participants

This study was carried out with a total of 18 adult healthy individuals (16 males and 2 females) with an age range: 18-30 years, and an average BMI of 26.6 ± 4.7. The experimental protocol was approved by Bahçeşehir University’s Ethics Committee and conducted according to the ethical principles of the Helsinki Declaration. Informed consent was obtained from all individuals participating in the study. Data acquisition The SESC technique requires the knowledge of joint angles and the horizontal CoM position during static postures. In order to measure joint angles of the subjects who were performing the assigned tasks, the Xsens Awinda system (Movella Inc., Henderson, NV, USA) was used. In static conditions, the CoP can be regarded as the horizontal CoM position^31^, hence a force platform (BERTEC FP-4060-05-PT) was used to record the CoP trajectory.

### Experimental protocol

Before starting the experiments, the participants were asked to put on the Awinda system using a lycra t-shirt, straps and the 17-sensors placed at their specified landmarks, starting with the head and followed by the shoulders, upper arms, forearms, hands, sternum, pelvis, upper legs, lower legs, and feet. Following the sensor placement, a calibration process was followed in order to adjust the sensors with the body of the participant. Afterward, the subject would stand on the force platform with their feet placed on specifically marked locations.

The SESC identification phase requires the recording of a number of joint configurations along with their respective CoP readings, hence the subjects were asked to perform a series of distinct postures which focused on the movement of the hips, knees, ankles, the L5/S1 and shoulder joints. The number of the performed joint configurations varied according to each subject’s flexibility and yielded an average of 98 postures across all participants. The selection of static postures was based on the criteria outlined in Gonzalez^32^. According to the specified conditions, a posture is deemed static if, within a 1 s window, the standard deviation (SD) of the angle measurements is below 1.5$^\circ$, and the SD of the CoP displacement is below 6 mm. In order to ensure that those criteria were perfectly met, the subjects were asked to maintain their balance in each posture for a minimum of 5 s.

### Data preprocessing and postprocessing

The CoP data, collected at 1000 Hz, from the force plate was filtered using a 4th order Butterworth filter with a 10 Hz cut-off frequency. Following the filtering process, the data was then resampled at 60 Hz to match the Xsens’ sampling frequency. The final preprocessing step involved merging the reference frames of both devices, Xsens and the force plate, into a unified global frame, using the L5 segment as the origin point, established at coordinates (0,0,0). This allows for the CoM estimated by the SESC to be comparable with the CoP reference, as they exist in a shared global frame. After completing these steps, the final dataset is divided into two separate datasets, with the first having the data of 75% of the postures, that are used to build the SESC model, and the rest of the data are later used for testing.

In adherence to the established procedure of SESC, we constructed four subject-specific SESC models for each participant, employing the aforementioned biomechanical models. These models were subsequently used to estimate the CoM location according to each posture present in the testing dataset.

### Statistical analysis

To evaluate the CoM estimations of all SESC models the root-mean-squared (RMS) error was calculated. We used SPSS (IBM ®SPSS ®Statistics 25, IBMCorp., USA) to investigate the differences between the estimation errors of each model. The normal distribution of the data was checked using the Shapiro-Wilk test. In the case of normality, the repeated measures analysis of variance (ANOVA) test was applied. As a post hoc test, the paired t-test was employed. For the non-normally distributed data, the non-parametric Friedman test was used, with the Wilcoxon signed-rank test applied as post hoc. The predefined significance level was determined as p < 0.05. To quantify the strength of the relationship between the estimations of each approach and the CoP readings, Pearson’s and Spearman’s correlation coefficients were calculated. To further investigate the accuracy and precision of each approach the Bland–Altman limits of agreement (LoA) method is used, along with a mixed-effects regression model to evaluate the existence of proportional biases. In this analysis, the agreement between the model and the gold standard reference is evaluated by determining the accuracy and precision of the tested method. The accuracy is determined by finding the mean difference (or bias) between the two sets of values (estimated and reference), while the precision is determined by calculating the LoA which represent 95% of the differences and tracking the spread of the measurements’ points with respect to those limits.

## Results

Following the data collection and preprocessing, for each participant, four subject-specific vectors were identified based on each model. These vectors were then applied to the testing postures in order to estimate the CoM. The results of each model were evaluated by comparing the CoM estimations to the ground truth values obtained from CoP static measurements along the anteroposterior (AP) and mediolateral (ML) axes.

**Figure 2:**
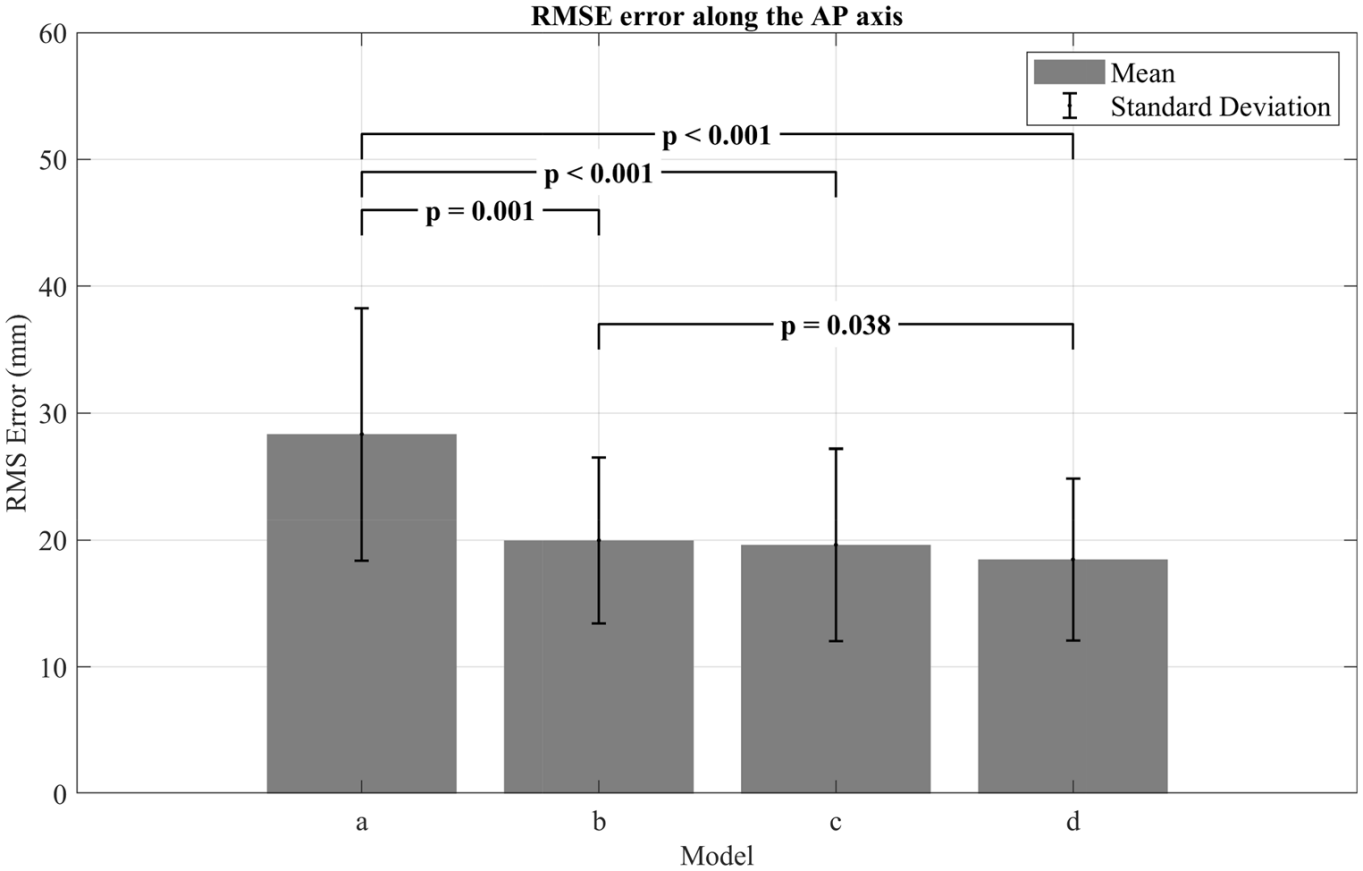
Comparison of the four SESC models along the AP axis based on RMS error and SD.

**Figure 3:**
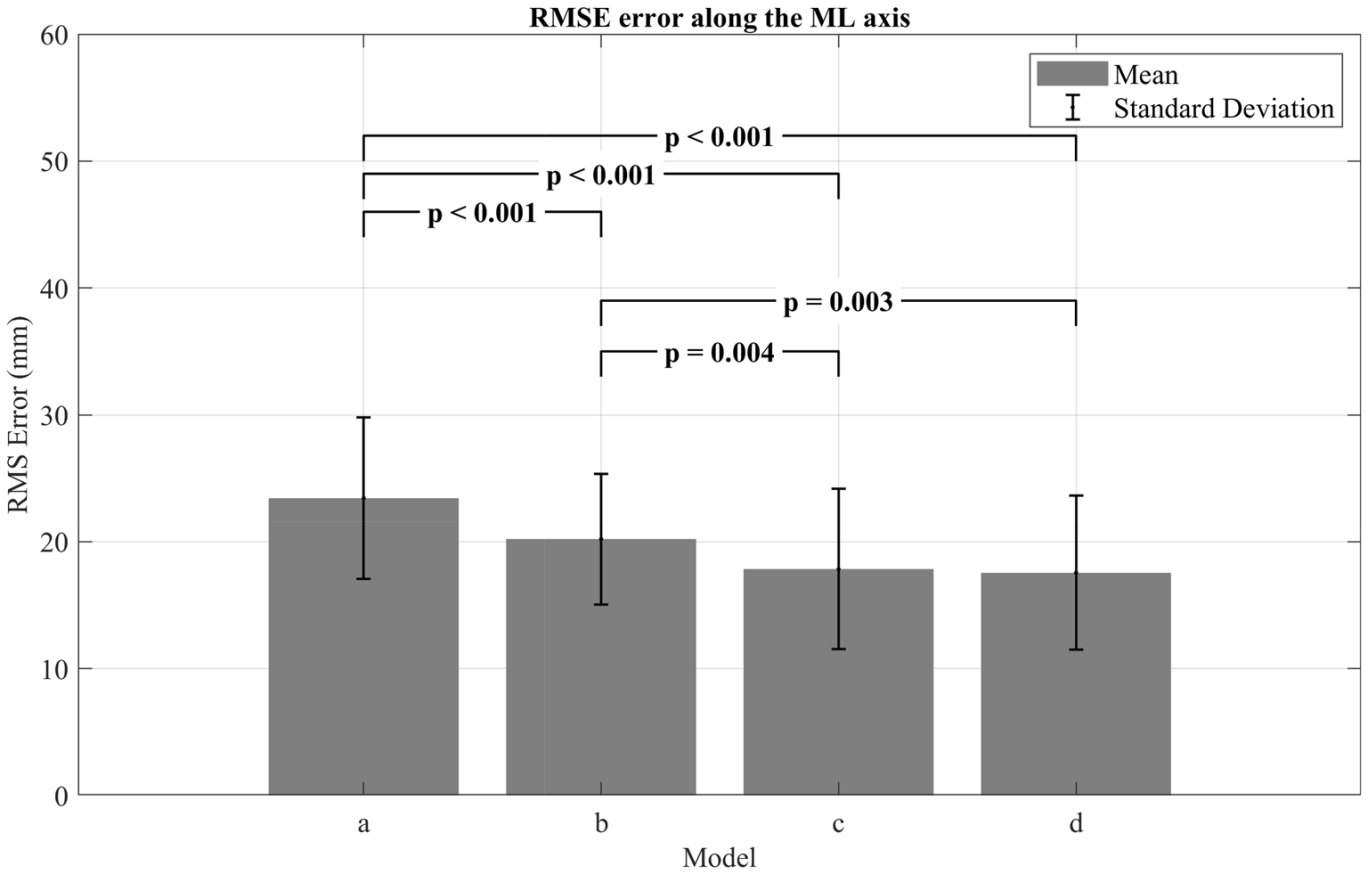
Comparison of the four SESC models along the ML axis based on RMS error and SD.

The RMS errors and their SDs are summarized in the bar graphs shown below (Figs. 2, 3). The mean RMS values for all models ranged between 8.2 and 51.8 mm along the AP axis, with an average of 28.3 (± 9.9) mm for model (a) with [25th percentile: 20.7, 75th percentile: 38.1], 20 (± 6.5) mm for model (b) with [25th percentile: 15.3, 75th percentile: 23.4], 19.6 (± 7.6) mm for model (c) with [25th percentile: 13.8, 75th percentile: 23.5], and 18.5 (± 6.4) mm for model (d) with [25th percentile: 13.7, 75th percentile: 22.4]. As for the ML axis, the RMS values ranged from 8.5 to 38.6 mm for all models with an average of 23.4 (± 6.4) mm for model (a) with [25th percentile: 20.6, 75th percentile: 24.7], 20.02 (± 5.1) mm for model (b) with [25th percentile: 17.7, 75th percentile: 22.0], 17.8 (± 6.3) mm for model (c) with [25th percentile: 11.8, 75th percentile: 21.6], and 17.5 (± 6.1) mm for model (d) with [25th percentile: 12.5, 75th percentile: 20.5]. With regard to the distribution of the RMS data in the results, it is important to highlight that non-normally distributed data was observed exclusively along the ML axis for models (a) and (d). As shown in Fig. 2, the RMS values of model (a) were significantly higher than those of the rest of the models (p < 0.05) along the AP axis, in addition to a statistically significant difference between the RMS values of model (b) and model (d) (p = 0.038). In contrast the pairwise comparison between the results of models (b) and (c) and the models (c) and (d) did not show any significant differences (p > 0.05). In Fig. 3, the RMS values along the ML axis were presented, and the results indicated a statistically significant difference between the results of model (a) and the rest of the models (p < 0.05). In addition, it is found that the RMS values of models (c) and (d) were both significantly lower than those of model (b) (p = 0.004 and p = 0.003, respectively).

As for the correlation coefficients presented in Table 1, the SESC estimations showed a strong correlation with the reference values along the AP axis with r ranging from 0.90 to 0.94 for all models. As for the ML axis, the estimations of models b, c, and d also showed strong correlations with the reference values (r ranging from 0.77 to 0.81), while the estimations of model (a) presented only a moderate correlation (r = 0.66) with the CoP values.

**Table 1:** Correlation coefficients for the CoP measurements and CoM estimations by the SESC models along the AP and ML axes.

Models	a	b	c	d
COP_AP	0.90*	0.93*	0.93*	0.94*
COP_ML	0.66*	0.77*	0.79*	0.81*

The values followed by ‘*’ indicate a p < 0.001.

In fact, correlation coefficient alone does not provide information related to how accurate or precise the estimations are, it only gives a clue about the strength of the linear relationship between the estimations and the ground truth values. For this reason, and also to conduct a comprehensive assessment of the models and determine the level of agreement between the estimated and reference values^33^, the Bland–Altman analysis was applied. The parameters used in the Bland–Altman assessment are summarized in Table 2.

**Table 2:** Bland–Altman parameters summary.

	Compared methods	Mean difference (fixed bias) in mm	LoA (lower to upper in mm	R-squared (p-value)	Regression equation
AP axis	Model (a) vs CoP	5.99	− 50.39 to 62.39	0.034 (< 0.001)	y = 0.08x − 1.23
	Model (b) vs CoP	0.23	− 37.60 to 38.07	0.004 (0.165)	y = − 0.02x + 2.29
	Model (c) vs CoP	0.26	− 38.91 to 39.42	0.005 (0.135)	y = − 0.02x + 2.35
	Model (d) vs CoP	0.01	− 35.24 to 35.25	0.003 (0.252)	y = − 0.01x + 1.51
ML axis	Model (a) vs CoP	− 7.24	− 48.81 to 34.33	1 (< 0.001)	y = − 2x + 42.36
	Model (b) vs CoP	− 5.13	− 42.44 to 32.17	0.395 (< 0.001)	y = − 0.83x + 16.47
	Model (c) vs CoP	− 3.14	− 36.34 to 30.07	0.022 (0.002)	y = − 0.11x + 0.30
	Model (d) vs CoP	− 2.56	− 35.19 to 30.06	0.021 (0.002)	y = − 0.11x + 0.70

**Figure 4:**
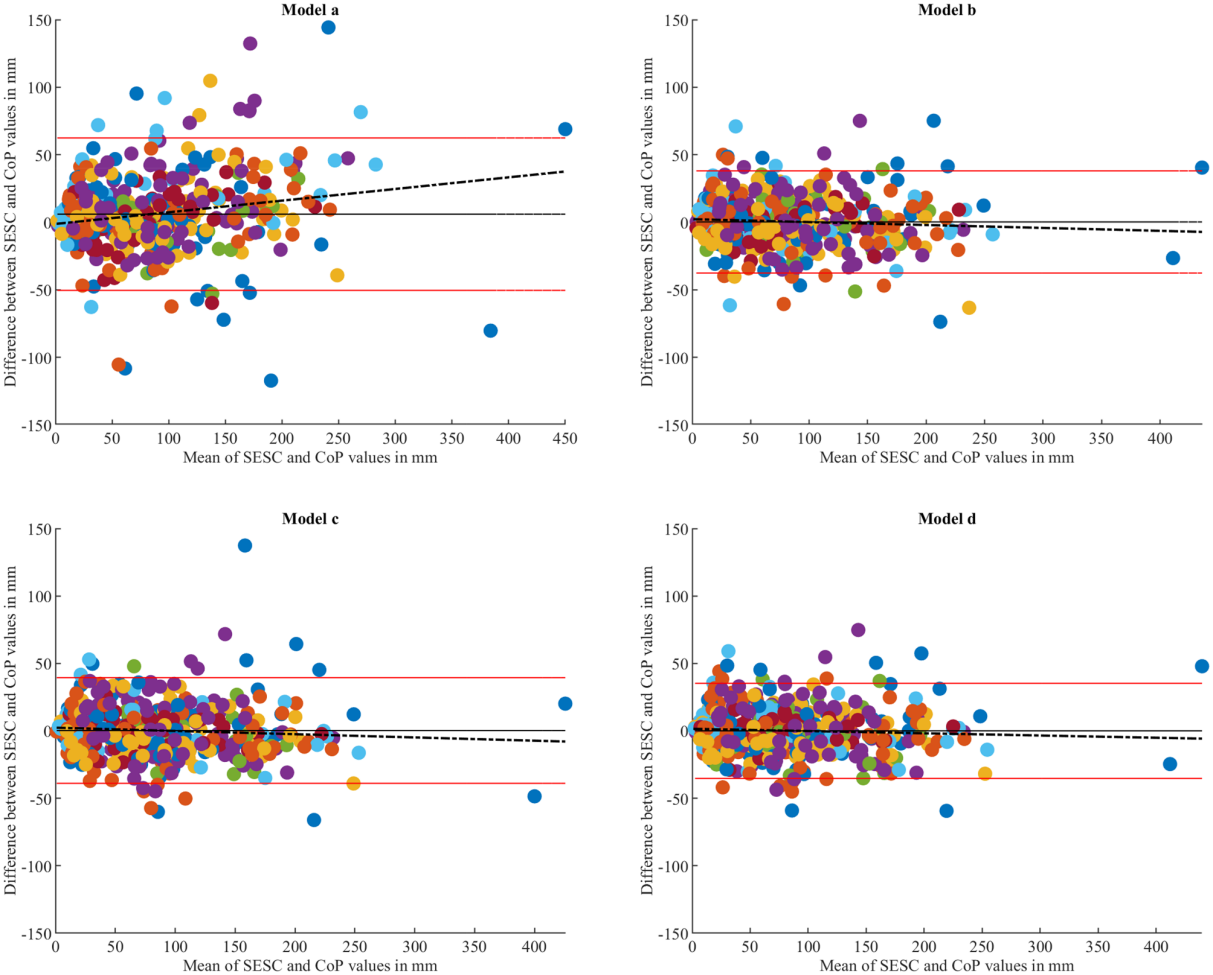
Bland–Altman plots illustrating the comparison between each model’s CoM estimations against the CoP readings along the AP axis. Each plot demonstrates the mean difference (black line) along with the LoA (red lines), indicating the range within which approximately 95% of the differences between predicted and reference values lie. The perfect agreement is indicated by a centered mean difference line with minimal spread between the LoA. In addition, a regression line is fitted with the data to quantify the existence of a trend within the data points. The colors of the dots correspond to each respective subject within the study.

Along the AP axis, the plots in Fig. 4 show that models (b), (c) and (d) have similar scattering patterns, in addition to having no noticeable fixed bias (mean difference ≈ 0 mm) and similar LoA ranges (± 37.8 mm, ± 39.16 mm and ± 35.2 mm, for models (b), (c) and (d), respectively). Furthermore, the regression analysis for these models showed non-significant R^2^ values (Table 2). On the other hand, the scattering pattern of model (a) measurements presents a noticeable fixed bias (mean difference = 5.99 mm) in addition to a wide LoA range (± 56.39 mm). The scattering pattern also reveals a visible trend that is described by the regression equation y = 0.08x − 1.23 (Table 2) with an R^2^ value of 0.034 (p < 0.001).

**Figure 5:**
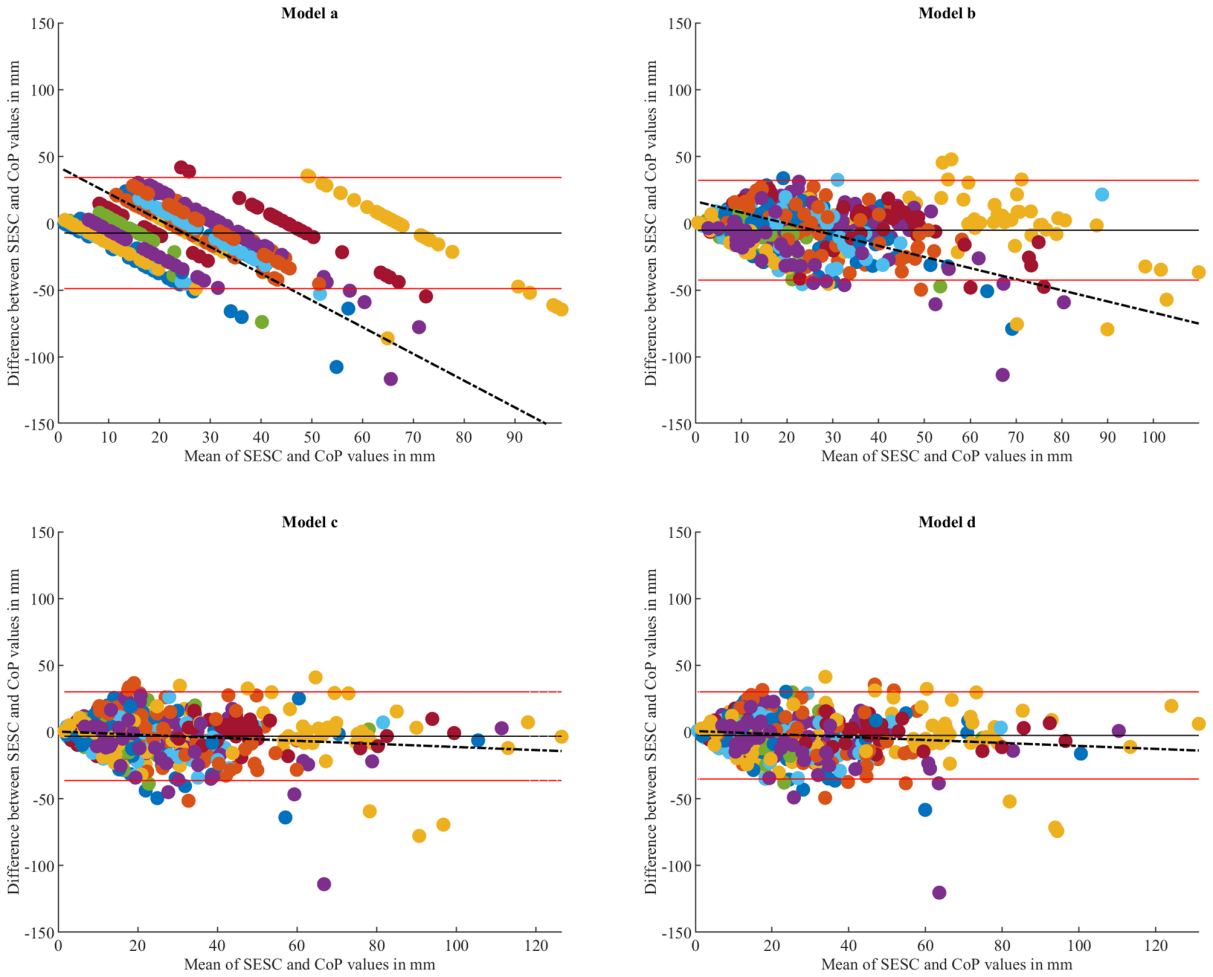
Bland–Altman plots illustrating the comparison between each model’s CoM estimations against the CoP readings along the ML axis. Each plot demonstrates the mean difference (black line) along with the LoA (red lines), indicating the range within which approximately 95% of the differences between predicted and reference values lie. The perfect agreement is indicated by a centered mean difference line with minimal spread between the LoA. In addition, a regression line is fitted with the data to quantify the existence of a trend within the data points. The colors of the dots correspond to each respective subject within the study.

As for the ML axis, the Bland-Altman plots (Fig. 5) presented similar scattering patterns for models (c) and (d) as they showed for the AP axis, with small, fixed bias values (− 3.14 mm and − 2.56 mm, respectively), in addition to acceptable LoA ranges (± 33.2 mm and ± 32.6 mm, for models (c) and (d) respectively). The regression analysis revealed a slight increase in R^2^ values (R^2^ = 0.022 and R^2^ = 0.021, for both models respectively) in comparison to the AP axis. As for the other two models, the scattering plot for model (a) shows 18 distinct linear patterns parallel to the line described by the regression equation y = − 2x + 42.36 (Table 2). This plot also revealed a fixed bias of − 7.24 mm and a LoA range of ± 41.5 mm. As for model (b), the scattering pattern shows that in comparison to the AP axis, the fixed bias is more deviated from the zero-line (mean difference = − 5.13 mm), while the LoA range remained close to the AP axis value (LoA range = ± 37.3 mm). Moreover, this scattering pattern also reveals a clear trend that is described by the equation y = − 0.83x + 16.47.

## Discussion

This paper proposes an evaluation of the SESC’s COM estimation technique which addresses the effect of the model complexity on the error range, accuracy and precision of the estimation. Four models were designed with each having a different combination of joints, segments and DoFs. The assessment of these models relied first on the evaluation of the error, measured in terms of RMS, difference between the estimations and the reference measurements—static CoP, in addition to determining the correlation between those values. The other part of the assessment utilizes the Bland–Altman analysis which quantifies the agreement between the estimations and the reference values, in addition to providing clear determination of the accuracy and precision of the models.

The least complex model (model a) is comprised of seven joints with each joint having only one DoF describing the flexion/extension rotation. In terms of the RMS evaluations, this model presented the largest error among all models (Figs. 1, 2) along both AP and ML axis (28.3 ± 9.9 mm and 23.4 ± 6.4 mm, respectively) (p < 0.05). Although this model had a larger RMS error along the AP axis, it presented a strong correlation with the CoP (r = 0.90), while its estimations along the ML axis showed only a moderate correlation with the reference values (r = 0.66). The Bland–Altman analysis for this model revealed fixed biases of 5.99 mm and − 7.24 mm along the AP and ML axes, respectively, which can be interpreted as a lack of accuracy according to Montenij et al.^33^. The dispersion of model (a)’s data points shown in Fig. 4 and the wide range of LoA highlight the lack of precision for this model. Moreover, a clear upward trend (proportional bias) was observed within the dispersion of the data, that was described by the regression equation y = 0.08x − 1.23 (Table 2) with an R^2^ value of 0.034 (p < 0.001). The existence of this trend indicates that as the estimation values get larger the model overestimates the CoM’s position along the AP axis. As for the ML axis, the plot (Fig. 5) shows that the data points follow a clear and perfectly fitted linear trend following a slope value of − 2. Such behavior can be attributed to the nature of the SESC calculations which rely on the pseudoinverse in one of the calculation steps. Since this model’s architecture does not include any DoF that allows for variations along the ML axis, the SESC predictions can only follow the linear model resulting from the pseudoinverse, hence the perfect linear shape of the data presented in the Bland–Altman plot.

Model (b) on the other hand, showed a significant improvement over model (a) in terms of RMS error along both AP and ML axes with respective mean error values of 20 ± 6.5 mm and 20.02 ± 5.1 mm. This improvement was also reflected in an increase with the correlation coefficients along both axes (Table 1), as well as a better agreement between the CoM estimations and the CoP readings along the AP axis, with narrower LoAs and absence of any biases or trends, as depicted in Fig. 4. On the contrary, the dispersion of the data for the ML axis shown in the Bland–Altman plots (Fig. 5) revealed a noticeable fixed bias of − 5.13 mm and a proportional bias indicated by the trend of the data scattering that follows the equation y = − 0.83x + 16.47 (Table 2) with a significant R^2^ value of 0.395 (p < 0.001). This behavior indicates that as the expected values get larger the model underestimates the actual values. Aside from the existence of these biases, this model’s results could be regarded as a significant improvement over the results of model (a).

Similar to model (b), model (c) presented a significant improvement over model (a) along both AP and ML axes in terms of RMS error and correlation coefficients, in addition to a significant improvement over model (b) along the ML axis. Although model (c) has two fewer joints than model (b), the increase in DoFs has given this model a significantly better estimation ability. The Bland–Altman plot shown in Fig. 5 (Model c) gives a clearer inspection of this improvement, as the estimations no longer present any fixed or proportional bias, but rather show a great agreement with the reference values.

The RMS values of the most complex model designed in this study, model (d), indicate no significant improvement over model (c). Although it had two additional joints (ankle joints) in comparison to the previous model, these joints did not contribute to improving the CoM estimation neither in terms of accuracy nor precision as depicted in the Bland–Altman plots. This can be attributed to the fact that the ankles are only responsible for the feet motion which represent only a small percentage of the total human body mass, thus making their inclusion in the model redundant and unnecessary.

Taking into consideration all the structural differences between the models and their respective estimation performances, it becomes evident that the DoFs play a pivotal role in determining the effectiveness of the estimation. This observation was particularly pronounced when comparing two models with same joints assignment but different joint ranges of motion, as in the case of models (b) and (d). However, it should be noted that while the difference between those two models in terms of DoFs had a statistically significant impact on estimation error, the average RMS difference of approximately 2 mm may not raise significant clinical concerns. In addition, the selection of specific joints also demonstrated a notable impact on the estimation quality. Models (a) and (c) serve as prime examples. Along the AP axis, the distinguishing factor was assigning the knee joint in model (c) instead of the ankle joint presented in model (a), which resulted in a significant RMS difference. On the contrary, the incorporation of an ankle joint to model (c) did not result in a significant RMS difference nor improved the agreement of the method with the reference as seen for model (d). These findings suggest that the proportion of mass which the joint is responsible for moving is closely intertwined with the CoM estimation, as the knee joint is known inherently handles higher mass percentage than the ankle joint. Consequently, this highlights the importance of diligent joint selection when designing a biomechanical human structure.

Aside from model complexity, it is noteworthy to highlight the fact that for the collection of the joint angles, used in CoM calculation, an IMU based mocap system was utilized. Such sensors are well known for being an appealing alternative for optical mocap system, especially for being more affordable and much easier to implement^34^. In spite of these sensors being convenient in many applications, utilizing a gold standard mocap device will no doubt provide an improvement in accurately estimating the human CoM. Therefore, when it comes to the selection of a mocap, researchers must understand the trade-offs associated with IMU sensors, as some applications may require a mocap with greater precision and fidelity. However, the recent advancements in IMU technology, more specifically in terms of High-Performance accelerometers^35^, are thought to significantly improve the performance of IMU based mocap systems in the future. It should be noted that the system used in this study, is regarded as a gold standard when it comes to IMU-based mocap technologies, and has been rigorously validated for use in biomechanical applications^36,37^. In addition to the type of equipment used in the study, several limitations should also be considered. First, the sample size of the subjects was relatively small, and the inclusion of a more extensive and diverse participant pool, including individuals with varying BMIs and a range of musculoskeletal and neurological disorders, could provide a more comprehensive assessment of the models’ performance. Additionally, the evaluation in this study primarily focused on static postures. Future work should prioritize assessing these models in dynamic situations to ensure their applicability in a broader range of scenarios.

Moreover, there remains a possibility for further investigation of SESC. Although the literature has presented numerous evaluation and enhancement techniques, such as mathematical simplifications^27,28^, the utilization of a Kalman filter for a dynamic identification of the SESC parameters^30^, and increasing the number of postures^27^, a comprehensive study incorporating a combination of these approaches, in conjunction with the model complexity effect, would provide profound insights into attaining the optimal modeling of the SESC technique.

## Conclusion

In this study, a comprehensive evaluation of the SESC CoM estimation method was presented. This evaluation focused on the most important step in SESC modeling, which is the human body’s biomechanical representation. Depending on the mocap system in use, one can either design a simple or a complex representation of the human body, by approximating the human segments as rigid bodies and its joints as typical mechanical joints. In this study, we came to the conclusion that up to a certain level of model complexity the quality of CoM estimation can significantly be improved. However, it is worth noting that depending on the application and task in question, less complex models are also capable of providing a sufficient estimation performance. This complexity/performance trade-off proves advantageous in scenarios constrained by hardware and temporal limitations.

### Supplementary Information

Supplementary Information.

## Data Availability

The authors declare that the data supporting the findings of this study are available upon reasonable request from the corresponding author and in accordance with applicable regulations and data usage agreements.
