# Supplementary material for: The effect of model complexity on the human center of mass estimation using the statically equivalent serial chain technique
Source: Sci Rep. 2023 Nov 20;13:20308. doi: 10.1038/s41598-023-47337-9 (PMC10662471; doi:10.1038/s41598-023-47337-9)
Supplement: Supplementary file 1 — Supplementary Information. [file 41598_2023_47337_MOESM1_ESM.pdf]

The effect of model complexity on the human center of mass estimation using the statically equivalent serial chain technique

Elie Chebel[1,*] **and Burcu Tunc**[2]

[1]Department of Computer Engineering, Bahcesehir University, Istanbul, 34353, Turkey
[2]Department of Biomedical Engineering, Bahcesehir University, Istanbul, 34353, Turkey
[*]elie.chebel@bahcesehir.edu.tr

Supplementary Information

In this study, subjects were asked to perform a variety of distinct postures, emphasizing movements of the hips, knees, ankles, the L5/S1, and shoulder joints. To facilitate the reproduction of results and provide a clearer understanding of the postures used, a selection of 27 posture images is included in this appendix. These images serve as representative samples of the postures performed by the subjects. The postures were designed to maximize the number of possible angle combinations, considering displacements of all upper and lower body segments. The number of postures can vary significantly due to the flexibility and anthropometric characteristics of each participant. While the maximum number of postures requested from each subject was 120, the average number of performed postures was around 99, reflecting the challenges some subjects faced due to certain postures requiring high levels of flexibility. The postures shown in the figures below were generated using the MVN Analyze (Movella Inc., Henderson, NV, USA, version: 2022.0.0). This appendix aims to provide a comprehensive visualized overview of the postures used in this study.

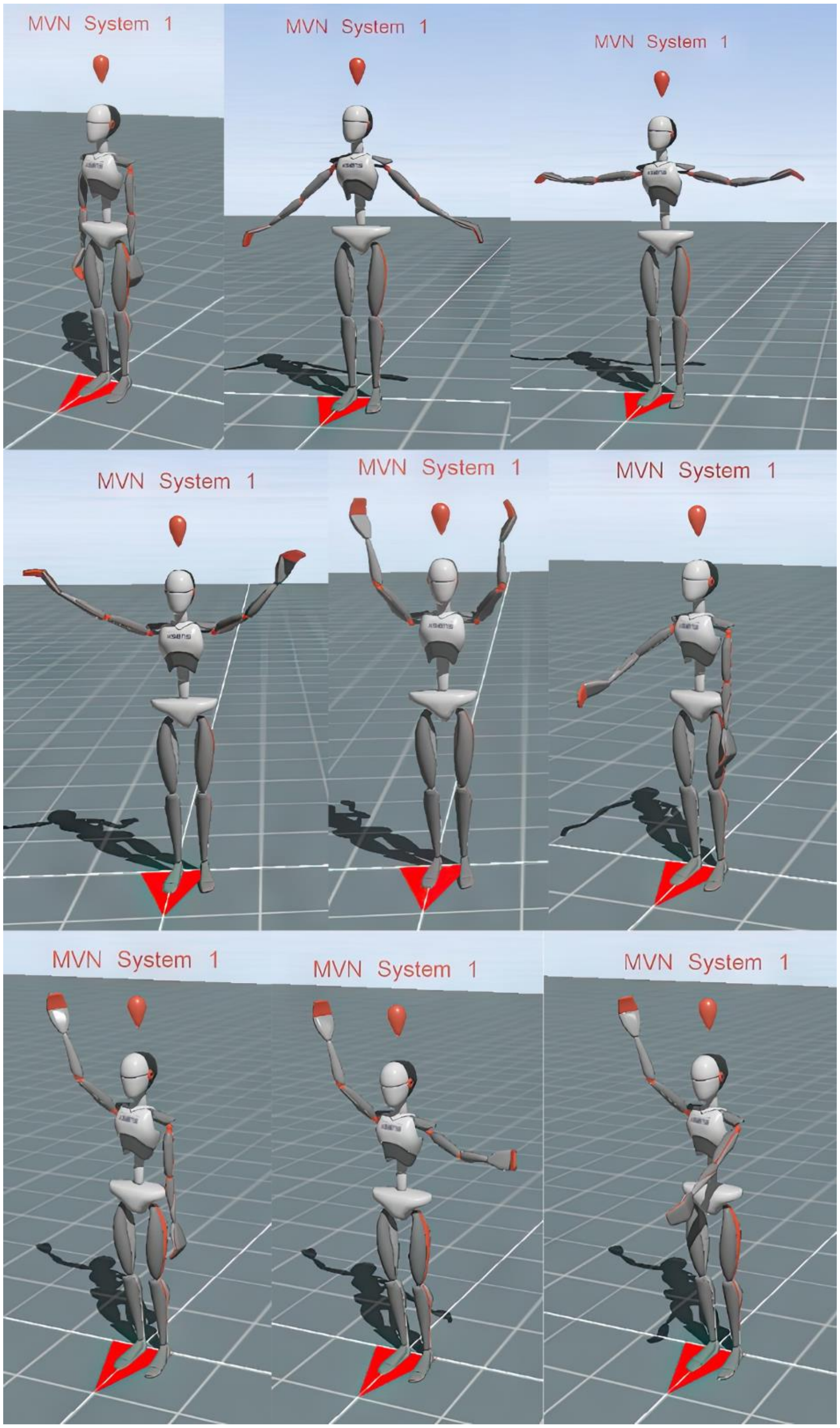

Figure S1. This figure shows a series of 9 different postures which focus mainly on the upper body motion. The images were generated using MVN Analyze (Version 2022.0.0, https://www.movella.com/products/motion-capture/mvn-analyze).

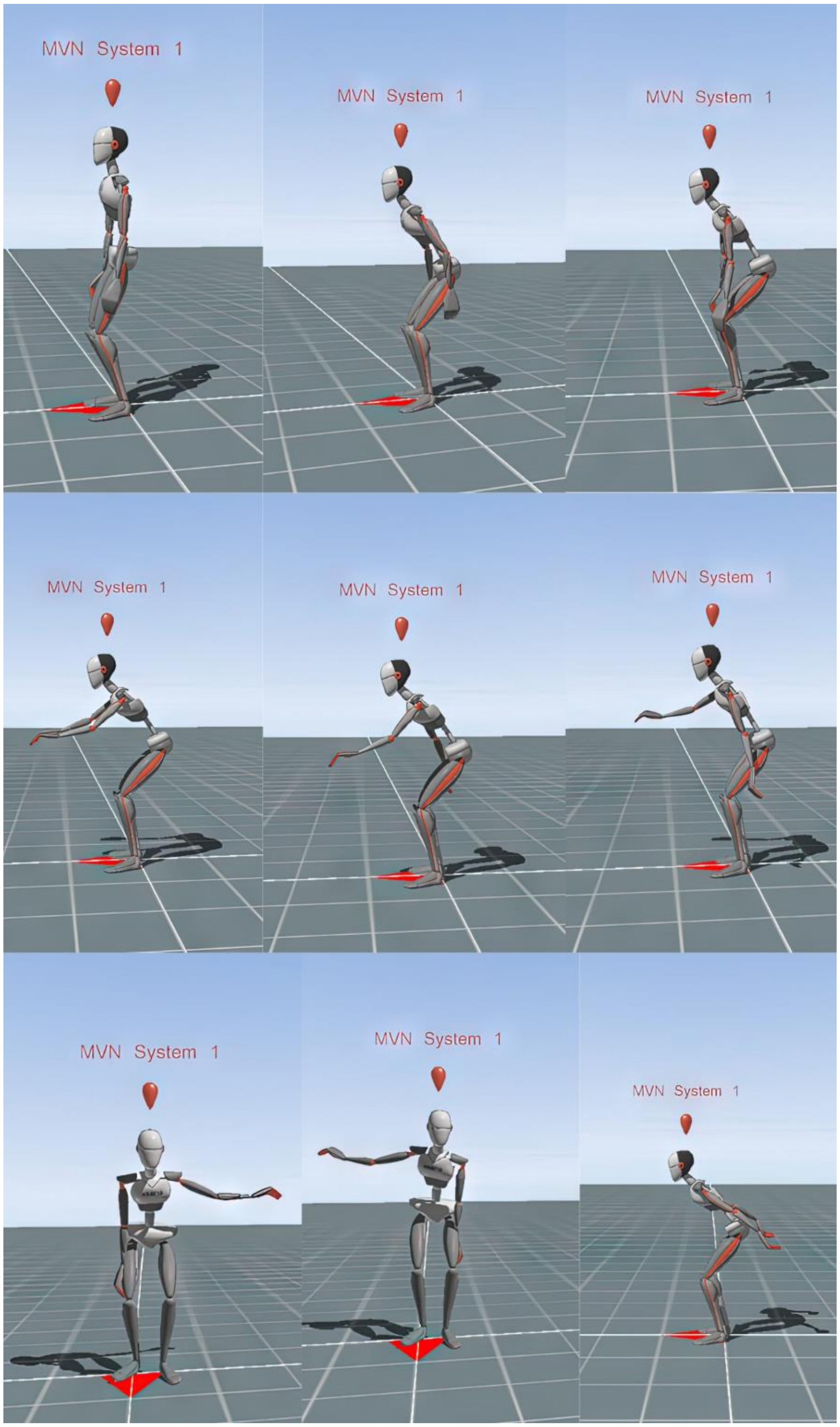

Figure S2. This figure shows a series of 9 different postures which focus on both the upper body and lower body motion. The images were generated using MVN Analyze (Version 2022.0.0, https://www.movella.com/products/motion-capture/mvn-analyze).

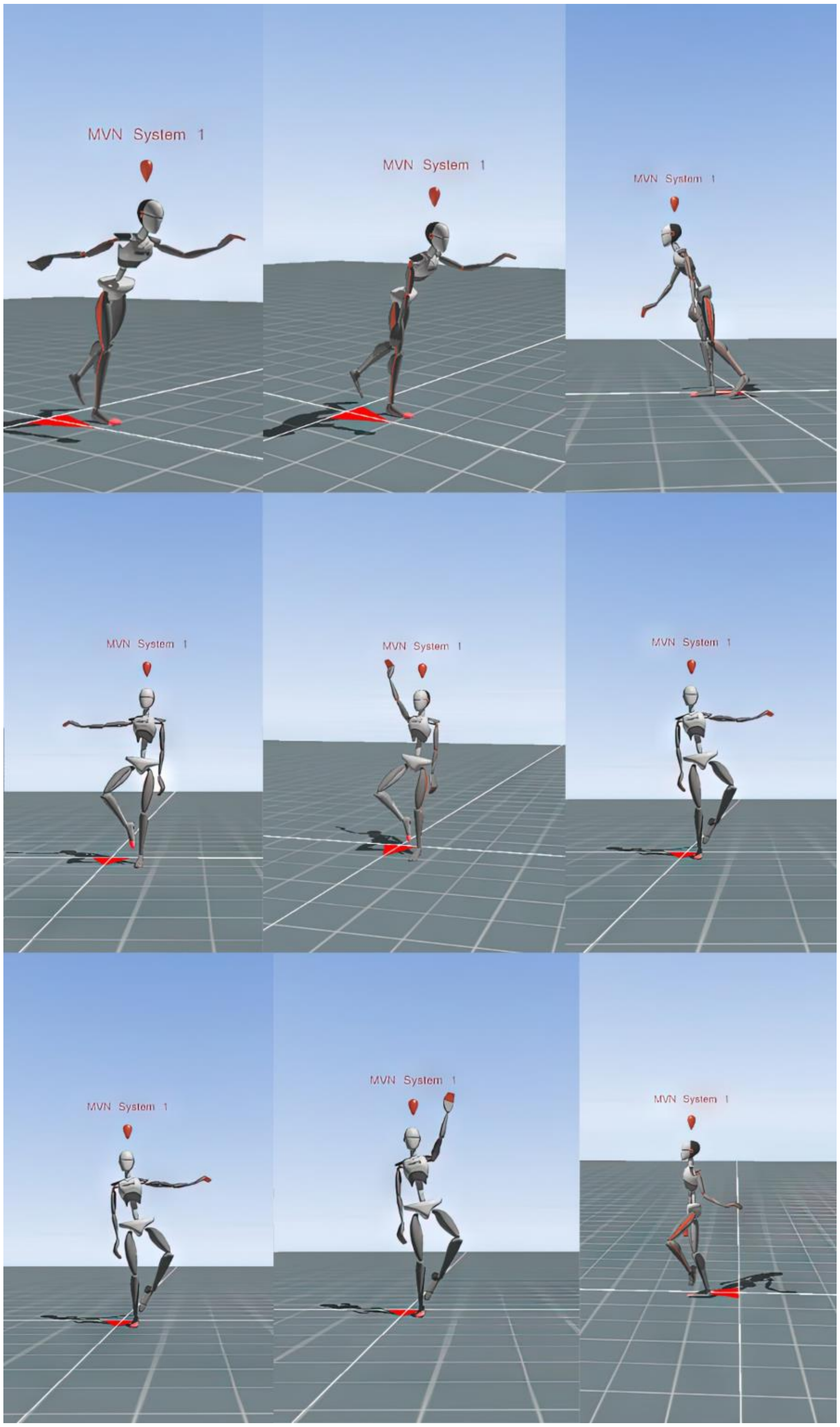

Figure S3. This figure shows a series of 9 different postures which focus on asymmetrical body postures. The images were generated using MVN Analyze (Version 2022.0.0, https://www.movella.com/products/motion-capture/mvn-analyze).
